# The Berlin Hepatitis C Manifesto: access to prevention, testing, treatment and care for people who use drugs

**DOI:** 10.1186/s41124-016-0021-9

**Published:** 2016-10-18

**Authors:** Eberhard Schatz, Katrin Schiffer, Mags Maher, Magdalena Harris, Xavier Major Roca, Mojca Maticic, Astrid Leicht

**Affiliations:** 1 Correlation Network, Amsterdam, The Netherlands; 2 European Network of People Who Use Drugs, London, UK; 3 Department of Social and Environmental Research, London School of Hygiene and Tropical Medicine, London, UK; 4 Programme on Substance Abuse, Public Health Agency of Catalonia, Autonomous Government of Catalonia, Barcelona, Spain; 5 Clinic for Infectious Diseases and Febrile Illnesses, University Medical Centre Ljubljana, Ljubljana, Slovenia; 6 Fixpunkt e.V., Berlin, Germany

**Keywords:** Drug User, Harm Reduction Service, Opioid Substitution Therapy, Berlin Declaration, Global Health Sector Strategy

## Abstract

The treatment of hepatitis C has entered a new era since the advent of curative pharmaceuticals. As policy, government and civil society assemble in response, there are still gaps to be addressed. The Manifesto on Hepatitis C and Drug Use, launched in Berlin during the Correlation Hepatitis C Initiative conference in October 2014, was formulated and endorsed by many key organizations in the hepatitis field. The Manifesto takes strides to pinpoint shortcomings in hepatitis action oriented towards the population most affected by the hepatitis C virus (HCV): active drug users.

Despite a considerable amount of evidence that active drug users are disproportionately affected by HCV, barriers to care remain. Engagement with representatives of communities of people who inject drugs (PWID) is imperative in order to effectively create guidelines which reflect reality. Unfortunately, widespread systemic stigmatization and lack of trust between affected communities, decision-makers and healthcare professionals have reproduced this divide. The Berlin Manifesto has identified a disconnect between evidence and action which must be answered.

In this roundtable discussion, experts from diverse parts of the hepatitis community have contributed their perspectives and experience on access to prevention, testing, and treatment for HCV in PWID. The authors discuss relevant topics such as realities of access to HCV treatment in the United Kingdom, interventions of a regional network of active drug users in Europe and lack of PWID involvement in government policy in Catalonia. Collectively they challenge the neglect of HCV in PWID by many decision-makers and health care professionals and promote a scale-up of integrated prevention and treatment strategies focusing on this population. The authors’ conclusions aim to clarify the discourse on hepatitis in order to prevent disease, save lives and work towards eventual hepatitis elimination.

## The aims of the Correlation Berlin Hepatitis C Manifesto: did dreams come true?

### Eberhard Schatz, Katrin Schiffer, Correlation Network, Amsterdam, The Netherlands

The Manifesto on Hepatitis C and Drug Use was launched in Berlin during the Correlation Hepatitis C Initiative conference in October 2014 [1]. Its development was a collaborative process involving several key experts from civil society. Additionally, it was endorsed by a broad range of leading organizations in the field, including the European AIDS Treatment Group, the European Association for the Study of the Liver (EASL), the European Liver Patients Association, Harm Reduction International, the International Network of People Who Use Drugs and the World Hepatitis Alliance.

The Manifesto is an urgent call to policy-makers and health care professionals to pay specific attention to the needs of the largest hepatitis C virus (HCV) patient group: people who inject drugs (PWID). This manifesto states that, despite evidence that treating (active) drug users is effective, and despite the fact that HCV treatment guidelines from EASL and WHO to the European Monitoring Centre for Drugs and Drug Addiction (EMCDDA) and European Centre for Disease Prevention and Control (ECDC) [2, 3, 4] recommend treatment for this target group, barriers to access remain. A Correlation survey pointed out that many countries in the European Union continue not to have hepatitis strategies and/or action plans, including measures for the treatment of PWID [5].

The Manifesto places HCV treatment in the broader context of integrated services and emphasizes the need to add harm reduction services to the cascade of treatment, including the safeguarding of full coverage and funding. Furthermore, it calls for the involvement of drug users not only in the execution of peer and prevention programs but also within decision-making processes like the development of programs. Above all, the Manifesto accentuates the complexities of stigma and discrimination due to the policy of criminalizing drug use(rs), which provides a major barrier to equal access to health services.

Overall, there is increased awareness of HBV and HCV, thanks to major efforts from a range of stakeholders at all levels. Importantly, there is a growing hepatitis advocacy community, making the quest to eliminate HCV even more imperative. In this context, the Berlin Manifesto plays an important role by highlighting the need to focus on people who use drugs and involve them in meaningful ways. One telling development is the increasing implementation of community and low-threshold testing programs, which reduce the barriers to testing and linkages to care, when deemed necessary. At the same time, other developments remain concerning: harm reduction and community service budgets are constantly cut or, in some settings, remain non-existent. It seems that the full integration of harm reduction services into the “treatment” cascade—which we prefer to call the continuum of care cascade—is one step too far for many local programs.

The fundamental question today is: after the launch of the Berlin Manifesto in 2014, and even more importantly, the introduction of highly efficient direct-acting antivirals since then, has the context of PWID and HCV improved? Understandably, the overall answer is complex. The contributions of this roundtable discussion provide insights from a range of experts, including a service provider, researchers, a hepatologist, a government official and, importantly, a representative from the European Network of People Who Use Drugs.

#### References

1. European Initiative on Hepatitis C and Drug Use. Manifesto. Berlin Declaration. Hepatitis C: access to prevention, testing, treatment and care for people who use drugs. Berlin, Germany. 23 Oct 2014. http://www.hepatitis-c-initiative.eu/index.php/component/content/article/2-uncategorised/29-manifesto-berlin-declaration. Accessed 12 Feb 2016.

2. European Association for the Study of the Liver. EASL recommendations of treatment for hepatitis C. J Hepatol. 2015;63:199-236. Accessed Feb 2016.

3. World Health Organization. Global Health Sector Strategies for HIV, Viral Hepatitis, STIs, 2016-2021. http://www.who.int/hiv/strategy2016-2021/en/. Accessed 12 Feb 2016.

4. EMCDDA/ECDC. ECDC and EMCDDA guidance. Prevention and control of infectious diseases among people who inject drugs, Stockholm, October 2011. http://www.emcdda.europa.eu/publications/ecdc-emcdda-guidance. Accessed 12 Feb 2016.

5. Maticic M, Videcnik Zorman J, Gregorcic S, Schatz E, Lazarus JV. Are there national strategies, plans and guidelines for the treatment of hepatitis C in people who inject drugs? A survey of 33 European countries. BMC Infect Dis. 2014;14(Suppl 6):14-26. doi:10.1186/1471-2334-14-S6-S14. Accessed Feb 2016.

## Drug users as activists: grassroots efforts to give voice to the drug using community

### Mags Maher, European Network of People Who Use Drugs, London, the United Kingdom

The European Network of People Who Use Drugs (EuroNPUD) is a regional network of drug user activists living across Europe who take drugs and represent the voice of Europe’s drug using community. A grassroots organization, we focus on drug-related activities that include human rights, drug policy reform, harm reduction, meaningful engagement and participation, peer/global advocacy, coordination, international cooperation, information, research and evaluation. We have also established communication systems among drug user groups and activists in the European Union (EU), which is key to our intelligence gathering and consultation at a grass roots level.

In short, EuroNPUD provides a vehicle for drug user activists and groups in the EU to work together to share best practices, provide mutual aid, offer peer-to-peer technical support and foster collective action and network development. Without active drug user involvement, no drug policy or project will accurately reflect what is happening within the drug using communities today.

In line with the European Hep C Initiative and their Berlin Manifesto: Hepatitis C: access to prevention, testing, treatment and care for people who use drugs (PWUD) [1], we have strongly recommended the development and implementation of European and national hepatitis C virus (HCV) strategies and action plans. We suggest that they include appropriately funded multidisciplinary approaches for HCV prevention and control among communities engaged in high-risk behaviors, including people who inject drugs. We have provided four southern European regions, Barcelona, Porto, Athens and Turin, with start-up grants to strengthen their drug user networks. The grants will focus on realistic and achievable interventions that influence hard to reach drug user groups to join the network as well as access the treatment interventions and facilities available to drug users in their regions.

An area that all four regions have strongly concentrated on is the testing of blood borne viruses (including HCV), which has proved very successful by using a peer-to-peer support model. This model allows drug users to work within drug services alongside professionals and provide interventions ranging from one-to-one support to drug users considering being tested, to collecting statistical information. The peer support workers, by default, have a non-judgmental approach to their community, which builds trust between those drug users accessing a service and the workers providing it. This platform allows our community members the opportunity to provide risk reduction information to their peers while working within a harm reduction framework; ultimately, we are the experts, and information is trusted when it comes directly from our community.

Using this model has resulted in a number of drug users being tested for HCV in North Turin, Italy, and Porto, Portugal. The experiences show, however, that there are still large numbers of PWUD who have not come forward for testing because of the stigma they experience when entering into a drug service, especially in such countries as Greece, where the prevalence of HCV and HIV continues to grow. We strongly think that prevalence declines if a peer support worker (drug user) is present but more research is needed to provide clear evidence.

Risk reduction messages are not infiltrating the drug using communities for a number of different reasons, primarily because Greece does not have a drug paraphernalia law and therefore cannot distribute injecting equipment; stigma toward drug users is still incredibly high across southern Europe; and women who are using drugs live in fear of losing their children, which discourages them from accessing services.

All four southern regions have identified huge gaps in service provision and are currently engaged in writing a report on drug users’ views on service provision in their region. EuroNPUD, in collaboration with Agência Piaget para o Desenvolvimento (APDES), PRAXIS, Reus University and Grupo de Ativistas em Tratamentos (GAT), is in discussions on developing a Diploma in Drug Dependency, to be called Project 20/20. The 6-month program will provide PWUDs with professional qualifications to work in the field of harm reduction without fear of prejudice or stigma. Having PWUDs work within drug services has already increased the number of drug users accessing services for several treatment interventions, including HCV testing, which will contribute toward eliminating HCV by 2030.

#### References

1. European Initiative on Hepatitis C and Drug Use. Manifesto. Berlin Declaration. Hepatitis C: access to prevention, testing, treatment and care for people who use drugs. Berlin, Germany. 23 Oct 2014. http://www.hepatitis-c-initiative.eu/index.php/component/content/article/2-uncategorised/29-manifesto-berlin-declaration. Accessed 30 April 2016.

## A revolution for all? Prioritizing HCV treatment access for people who inject drugs in England

### Magdalena Harris, Department of Social and Environmental Research, London School of Hygiene and Tropical Medicine, London, the United Kingdom

In 2010, the United Kingdom (UK) achieved the unenviable ranking of 12th among 14 comparable counties for HCV treatment provision [1], with less than 3 % of those diagnosed accessing HCV treatment per year [2]. Of the 3 %, very few were people who inject drugs (PWID). Despite a “pharmacological revolution” in HCV treatment options [3], England’s position on the world treatment access stage is unlikely to change.

2015 has been characterized by dispute and delay in regard to all-oral direct-acting antiviral (DAA) access in England. Although expanded DAA access has been recommended and deemed cost-effective by the National Institute for Health and Care Excellence (NICE) [4], NHS England disputes the affordability of this strategy in a context of fiscal austerity, instead advocating strategies of “watchful waiting” and “treatment sequencing” [5]. This approach is in stark contrast with those advocated by neighboring countries Scotland, Portugal, France and Spain, which, in line with an elimination approach, have scaled up DAA access. NHS England’s affordability concerns have set the stage for a conservative prioritization of treatment access by the 22 Operational Delivery Networks (ODN) tasked with this role. As of February 2016, DAA access is ostensibly extended to all people with genotype 1 and 4, yet budget-related “run-rate” 2 restrictions mandate each ODN to prioritize treatment according to “clinical need” [4]. This circumspect policy landscape is unlikely to improve HCV treatment access for PWID—particularly in the absence of priority population treatment targets.

Given the UK’s prior record of treatment provision for PWID, priority population targets may be required to attain equity of access. Guidelines are not enough. A 2010 audit of UK hospitals found that many follow formal or informal policies restricting HCV treatment access for PWID [6]. Recent qualitative data (see [7] for methods) capture this inequity:

The criterion for treatment at [hospital] is no drug use. Within outreach it was stable drug use 2 to 3 times a week. I don’t think that’s even the problem. I think the problem is getting them to come. Because they don’t want to come to secondary care. And there’s a wait for appointments, so I think they’re likely to DNA (“do not attend”). (Clinical Nurse Specialist)

Notable is how the hospital’s access policy of “no drug use,” contravening UK and European guidelines, is minimized and rationalized in regard to the perceived unreliability and disinterest of PWID. Pervasive discrimination in the hospital environment, such as treatment access dependent on injecting status, has a recursive effect by rationing local treatment expectation and, thus, disabling demand [8]. Outreach models, while ideal in theory, can be used to rationalize treatment refusal for PWID in mainstream care, regardless of the availability of local outreach provision. When the above outreach service closed due to funding restrictions, PWID in the districts were bereft of treatment options. For those unable to demonstrate treatment “readiness” and “stability” (drug use 2–3 times a week), even outreach was not an option.

Given this context, it is unclear how the benefits of DAA treatment regimens will affect the lives of PWID. The elimination vision has been the most successful in facilitating the rehabilitation of PWID as a treatment-viable population. While it is regrettable that 1) this rehabilitation is needed and 2) that it is afforded by population-health incidence reduction goals rather than those based on human rights [9], the reluctance of NHS England to engage with this vision augers poorly for the attainment of the Berlin Manifesto [10] goals, particularly regarding treatment access for PWID.

#### References

1. Richards M. Extent and causes of international variations in drug use. A report for the Secretary of State for Health. London: COI; 2010.

2. Public Health England. Hepatitis C in the UK: 2014 Report. London: Public Health England; 2014.

3. Zoulim F, Liang T, Gerbes A, Aghemo A, Deuffic-Burban S, Dusheiko G, et al. Hepatitis C virus treatment in the real world: optimising treatment and access to therapies. Gut. 2015;64:1824–1833.

4. National Institute for Health and Clinical Excellence. Ledipasvir—sofosbuvir for treating chronic hepatitis C. NICE technology appraisal guidance [TA363]. 2015. http://www.nice.org.uk/guidance/ta363/chapter/1-guidance. Accessed 15 Dec 2015.

5. NHS England submission to questions raised by NICE following consultation responses to hepatitis C drug appraisals. Confidential document. 2015.

6. All-Party Parliamentary Hepatology Group. In the dark: an audit of hospital hepatitis C services across England. London: All-Party Parliamentary Hepatology Group; 2010.

7. Harris M, Ward E, Gore C. Finding the undiagnosed: A qualitative exploration of hepatitis C diagnosis delay in the United Kingdom. J Viral Hepatol. 2016; 23:479-486. doi:10.1111/jvh.12513.

8. Rhodes T, Harris M, Martin A. Negotiating access to medical treatment and the making of patient citizenship: the case of hepatitis C treatment. Sociol Health Ill. 2013;35:1023–1044.

9. Wolfe D, Luhmann N, Harris M, Momenghalibaf A, Albers E, Byrne J, Swan T. Human rights and access to hepatitis C treatment for people who inject drugs. Int J Drug Policy. 2015;26:1072–1080.

10. European Initiative on Hepatitis C and Drug Use. Manifesto. Berlin Declaration. Hepatitis C: access to prevention, testing, treatment and care for people who use drugs. Berlin, Germany. 23 Oct 2014. http://www.hepatitis-c-initiative.eu/index.php/component/content/article/2-uncategorised/29-manifesto-berlin-declaration. Accessed 15 Dec 2015.

## Failing to involve people who inject drugs in hepatitis C policy definition and implementation in Catalonia

### Xavier Major Roca, Programme on Substance Abuse, Public Health Agency of Catalonia, Autonomous Government of Catalonia, Spain

The Berlin Declaration: Hepatitis C: access to prevention, testing, treatment and care for people who use drugs [1] correctly emphasizes the issue that is key to addressing the hepatitis C (HCV) epidemic in Europe: the involvement of the most affected population, people who inject drugs (PWID), in all stages of policy formulation and implementation.

But this does not happen often. I will explain some situations that have taken place in Catalonia, Spain, which I suspect might occur in other European countries:

1. In 2015, an HCV strategy was drawn up by the Catalan government, with the participation of the main patient association, which, surprisingly, does not have any drug user members.
2. The Department of Health has been providing guidance to clinicians on access to HCV treatment, but the guidance does not include specific criteria related to PWID [2]. As a result, inclusion of drug users in treatment still frequently depends on an individual doctor’s attitudes toward drug users. In Catalonia, most coinfected (with HIV) people are treated by internists, who are much more likely than hepatologists (who treat monoinfected patients) to provide HCV treatment to coinfected PWID. Internists, in contrast to hepatologists, have been dealing with drug users since the first outbreak of the HIV epidemic over three decades ago, so they have learned through experience how to care for drug users in an effective and nondiscriminatory way. Attitudes toward drug users can greatly impinge on access rates. Clear and specific criteria on drug users’ access to treatment, such as those mentioned in the European Association for the Study of the Liver (EASL) [3] and WHO [4] guidelines, are needed in Catalonia, but the involvement of drug users, including lobbying, is paramount in order to ensure that these guidelines are established.
3. In our consumption rooms, many HIV-positive active drug users are on antiretroviral therapy, even though they continue to inject drugs regularly, but few active drug users infected with HCV have ever been on HCV antiviral treatment. HIV-positive drug users have benefited from the strong lobbying efforts of influential groups and individuals to promote easy and equitable access to HIV medication. Drug users who are infected with HCV, however, have not had the same experience because they are not as well represented by influential groups as HIV-positive drug users are, and they also still suffer from stigma within the general community, which can affect their access to treatment and, ultimately, their health outcomes. These disparities might not exist if drug users had been able to mobilize a committed group of effective stakeholders as support. Additionally, possibly also a result of a lack of effective lobbying efforts on the part of active drug users, the Department of Health may not have known any representatives from the drug user community to contact for advice regarding the needs of these individuals.

These and other examples illustrate the most serious issues confronting PWID in Catalonia—and possibly throughout other European countries. In most countries, drug user organizations are often not well functioning, in large part because their members have difficult lives and have little experience in capacity building and governance. As a result, it is up to governments and other members of civil society to engage them in helping develop appropriate policies and programs to ensure that their needs are met. Any health strategy should, without a doubt, take into account the views and experiences of the main target population if it wants to succeed.

One way to address the needs of PWID effectively is to train hepatologists on drug use, and support them when they treat drug users. Involving drug users directly in this training is critical. This approach is a crucial means of improving linkages to care and, consequently, increasing treatment uptake. Shared care between drug care centers and liver specialists is a key issue, and a new initiative in Copenhagen is a promising model for the rest of Europe [5].

In 2015, Catalonia made progress toward realizing the goals of the 2014 Manifesto by establishing a strategy that includes clear measures to reduce HCV among PWID. While this was an important step, Catalonia still has made no progress in seeking input from drug users; implementation of the strategy, in general, has been hampered by the economic crisis and current drug prices; and treatment lags behind.

These constraints notwithstanding, strides forward can be seen: prevention campaigns such as the commemoration of World Hepatitis Day have been implemented in drug facilities, and in 2015, Spain took part in the European HIV testing week, which included hepatitis C for the first time. And, importantly, additional steps have been taken to further the prevention, treatment and care for HCV infected drug users. A hepatitis C rapid test is now routinely provided by harm reduction services in Catalonia, and in 2016, research will be carried out on an antigen test with dried blood spot technology. Guidelines have been established for needle and syringe programs and include recommendations such as: 1) sterile equipment should be provided based on client needs without further restrictions, and 2) PWID should be systematically encouraged to take with them all injection paraphernalia that they need, along with sterile syringes.

#### References

1. European Initiative on Hepatitis C and Drug Use. Manifesto. Berlin Declaration. Hepatitis C: access to prevention, testing, treatment and care for people who use drugs. Berlin, Germany. 23 Oct 2014. http://www.hepatitis-c-initiative.eu/index.php/component/content/article/2-uncategorised/29-manifesto-berlin-declaration. Accessed 7 Apr 2016.

2. Catsalut. Dictamen sobre el tractament de pacients adults amb hepatitis c crònica. 16 April 2015. http://catsalut.gencat.cat/ca/detalls/articles/article_tractament_hepatitis_C_cronica. Accessed 7 Apr 2016.

3. European Association for the Study of the Liver. EASL recommendations on treatment of hepatitis C 2015. J Hepatol. 2015;63:199–236.

4. WHO. Guidelines for the screening, care and treatment of persons with hepatitis C infection. http://www.who.int/hiv/pub/hepatitis/hepatitis-c-guidelines/en/. Accessed 5 May 2016.

5. Centre for Health & Infectious Disease Research (CHIP). SACC Project (Shared Addiction Care Copenhagen). Copenhagen, Denmark. http://www.chip.dk/Collaborations/SACC. Accessed 7 Apr 2016.

## Hepatitis C treatment for all PWID: myth or reality?

### Mojca Maticic, Clinic for Infectious Diseases and Febrile Illnesses, University Medical Centre Ljubljana, Slovenia

The Berlin Manifesto on HCV and Drug Use calls for countries to fund, support and implement hepatitis C virus (HCV) treatment and care services as part of their comprehensive national healthcare policies. The Manifesto also urges pharmaceutical companies and European Union member states to reach agreement on reducing the price of new medications and allow the scale-up of treatment [1].

In Europe, three million HCV infected people inject drugs, with the HCV seroprevalence ranging between 5–90 % [2]. In the last few years, the vast majority of newly infected people reported injecting drug use as the mode of infection [3, 4]. In the current era of highly effective, safe and patient-friendly HCV treatment, speculation is directed toward the elimination of HCV infection in the near future [5–7]. Unfortunately, treatment of HCV in PWID has long been the subject of controversy, although both adherence to treatment and degree of treatment efficacy among PWID have been shown to be comparable to those among non-PWID, even in the era of pegylated interferon (PegIFN) plus ribavirin, which a modeling study [8–10] proved to enhance prevention of HCV transmission.

Until 2014, when the Berlin Manifesto on HCV and Drug Use was launched as part of the European HCV Initiative [1], key data on HCV management and on the largest HCV infected group in Europe were sparse and suggested that only a negligible percentage of either current or former PWID from high-income countries were treated for HCV [11]. Published studies, for example, have shown that the percentage of these current or former PWID being diagnosed and entering HCV treatment ranged from 0.9 to 18 % (median 9.5 %, IQR 3.5–15) [12]. Another systematic review of literature within the WHO European region pointed to considerable knowledge gaps regarding treatment uptake levels within this population, suggesting that there may be major differences between and within countries in relation to treatment availability, drug-using populations in need of treatment and the existence of integrated healthcare services targeting drug users [13]. For example, reports have shown that various HCV treatment models have been used across different settings, with varying degrees of efficacy achieved. [14–16]. Most importantly, data reported in 2014 from 33 European countries revealed that there was a lack of existing national strategies, action plans and clinical guidelines on HCV treatment for PWID in several European countries, with only 27 % of them reporting that active drug users had access to HCV treatment [17].

As they have struggled to overcome barriers to HCV treatment for PWID, cohorts of untreated HCV-infected PWID have been aging, with a significant proportion of untreated PWID developing cirrhosis, and even hepatocellular carcinoma, at 20–30 years post-infection [18, 19]. Up to 25 % of deaths among PWID have already been connected to HCV infection [18]. Treatment options have dramatically improved, however, so urgent action is needed to increase access to treatment and optimize the circumstances for its uptake in PWID.

Several updated international treatment guidelines call for HCV treatment for PWID, primarily for two reasons: to prevent life-threatening diseases on the individual level and—using a public health approach—to prevent the further spread of HCV infection. The 2015 European Association for the Study of the Liver (EASL) guidelines regard treatment for active HCV-infected PWID as a priority due to the risk of their transmitting HCV [20]. The 2015 International Network for Hepatitis in Substance Users (INHSU) recommendations for the management of HCV infection in PWID demonstrated that HCV treatment among PWID is feasible and provided a framework for HCV assessment and care in this group [21]. High prices of the new direct-acting antivirals, however, present the largest barrier to expanding HCV treatment among PWID in the majority of European countries. Yet, in spite of this, some countries, including Iceland, Portugal, Scotland and Spain have negotiated lower prices and initiated elimination campaigns.

To overcome remaining barriers, stronger national HCV treatment policies are needed, along with efforts to increase knowledge and reduce misconceptions among physicians regarding the feasibility and importance of treating HCV infected PWID. Treating HCV in PWID will save lives, reduce the potential disease burden in the future and lower costs to the healthcare systems in the long run. And, importantly, it will also be a crucial first step toward elimination of HCV.

#### References

1. European Initiative on Hepatitis C and Drug Use. Manifesto. Berlin Declaration. Hepatitis C: access to prevention, testing, treatment and care for people who use drugs. Berlin, Germany. 23 Oct 2014. http://www.hepatitis-c-initiative.eu/index.php/component/content/article/2-uncategorised/29-manifesto-berlin-declaration.

2. Hahne SJ, Veldhuijzen IK, Wiessing L, Lim TA, Salminen M, Laar M. Infection with hepatitis B and C virus in Europe: a systematic review of prevalence and cost-effectiveness of screening. BMC Infect Dis. 2013;13:181-98.

3. Esteban JI, Sauleda S, Quer J. The changing epidemiology of hepatitis C virus infection in Europe. J Hepatol. 2008;48:148-62.

4. European Monitoring Centre for Drugs and Drug Addiction (EMCDDA): European Drug Report 2013: trends and development. Lisbon, Portugal. May 2013.

5. World Hepatitis Summit. Glasgow Declaration on Viral Hepatitis. Glasgow, Scotland. 2–4 Sept 2015. http://www.worldhepatitisalliance.org/en/glasgow-declaration-viral-hepatitis-filipino.

6. HCV Brussels Summit. Elimination Manifesto. Brussels, Belgium. 17 Feb 2016. http://www.hcvbrusselssummit.eu/elimination-manifesto.

7. World Health Organization. Global health sector strategies for HIV, viral hepatitis, STIs, 2016-2021. http://www.who.int/hiv/strategy2016-2021/en/.

8. Aspinall EJ, Corson S, Doyle JS, Grebely J, Hutchinson SJ, Dore GJ, et al. Treatment of hepatitis C virus infection among people who are actively injecting drugs: a systematic review and meta-analysis. Clin Infect Dis. 2013; 57(Suppl 2):80-9.

9. Dimova RB, Zeremski M, Jacobson IM, Hagan H, Des Jarlais DC, Talal AH. Determinants of hepatitis C virus treatment completion and efficacy in drug users assessed by meta-analysis. Clin Infect Dis. 2013;56:806-16.

10. Martin NK, Vickerman P, Foster GR, Hutchinson SJ, Goldberg DJ, Hickman M. Can antiviral therapy for hepatitis C reduce the prevalence of HCV among injecting drug user populations? A modeling analysis of its prevention utility. J Hepatol. 2011;54:1137-44.

11. Grebely J, Raffa JD, Kraiden M, Kerr T, Fischer B, Tyndall MW. Low uptake of treatment for hepatitis C virus infection in a large community-based study of inner city residents. J Viral Hepat. 2009;16:352-38.

12. Wiessing L, Ferri M, Grady B, Kantzanou M, Sperle I, Cullen KJ, et al. Hepatitis C virus infection epidemiology among people who inject drugs in Europe: a systematic review of data for scaling up treatment and prevention. PLoS One 2014;9:e103345.

13. Lazarus JV, Sperle I, Maticic M, Wiessing L. A systematic review of hepatitis C virus treatment uptake among people who inject drugs in the European Region. BMC Infect Dis. 2014;14(Suppl 6):S16.

14. Hellard M, Sacks-Davis R, Gold J. Hepatitis C treatment for injection drug users: a review of the available evidence. Clin Infect Dis. 2009;49:561-73.

15. Zanini B, Covolo L, Donato F, Lanzini A. Effectiveness and tolerability of combination treatment of chronic hepatitis C in illicit drug users: meta-analysis of prospective studies. Clin Ther. 2010;32: 2139-59.

16. Bruggmann P, Litwin AH. Models of care for the management of hepatitis C virus among people who inject drugs: one size does not fit all. Clin Infect Dis. 2013 57(Suppl 2):56-61.

17. Maticic M, Videcnik Zorman J, Gregorcic S, Schatz E, Lazarus JV. Are there national strategies, plans and guidelines for the treatment of hepatitis C in people who inject drugs? A survey of 33 European countries. BMC Infect Dis. 2014;14(Suppl 6):14-26.

18. John-Baptiste A, Krahn M, Heathcote J, Laporte A, Tomlinson G. The natural history of hepatitis C infection acquired through injection drug use: meta-analysis and meta-regression. J Hepatol. 2010;53:245-51.

19. Smith DJ, Combellick J, Jordan AE, Hagan H. Hepatitis C virus (HCV) disease progression in people who inject drugs (PWID): a systematic review and meta-analysis. Int J Drug Policy. 2015; 6:911-21.

20. European Association for the Study of the Liver. EASL recommendations of treatment for hepatitis C. J Hepatol. 2015;63:199-236.

21. Grebely J, Robaeys G, Bruggmann P, Aghemo A, Backmund M, Bruneau J, et al. Recommendations for the management of hepatitis C virus infection among people who inject drugs. Int J Drug Policy. 2015;26:1028-38

## Effective prevention of hepatitis C among people who inject drugs

### Astrid Leicht, Fixpunkt e.V., Berlin, Germany

The Berlin Manifesto on Hepatitis C and Drug Use [1] calls for the scale-up of harm reduction and, in particular, opioid substitution therapy (OST) and needle and syringe programs (NSP). Taking into consideration the easy transmission of hepatitis C (HCV) in comparison to HIV, the Manifesto highlights the need to ensure higher quality standards and coverage for harm reduction services in order to prevent HCV.

Needle and syringe programs and OST, along with the provision of comprehensive information, education and HIV/HCV pre/post-test counseling, are recommended interventions by WHO, UNAIDS, the United Nations Office on Drugs and Crime (UNODC) [2], the European Centre for Disease Prevention and Control (ECDC) and the European Monitoring Centre for Drugs and Drug Addiction (EMCDDA) [2, 3, 4]. Nevertheless, in most European countries, NSP remain in the shadows, neglected by national officials and international funders, as evidenced by the fact that the number of programs (coverage) is often too low in most European countries [6] as well as throughout the rest of the world [5], if they exist at all. In some instances, NSP are given low priority even by drug use treatment organizations.

One new development impacting prevention services is the treatment as prevention paradigm, which emphasizes the cure, rather than prevention of HCV infections. This scenario engages people who inject drugs (PWID) in their own treatment and cure. This groundbreaking development is positive, but it also has a “perverse” effect, in that, some infected individuals who have been treated and cured might begin to feel safe and therefore may risk reinfection if they do not use clean needles and syringes, or move from injecting drugs to OST. Some harm reduction organizations appear to overlook the treatment as prevention approach, and instead invest in prevention activities that are considerably less expensive —and potentially less effective—than direct-acting antivirals (DAAs).

Scientific experts and practitioners working in the field of harm reduction have not discussed interventions that might be more effective in preventing both HIV and HCV even though such discussions have taken place around medical treatments (OST, DAA) and psychosocial interventions. An exception is research related to low dead-space (LDS) syringes and needles. WHO has, in fact, rated evidence in this field as being of “very low quality” [5]. We therefore are in serious need of in-depth investigations into interventions that might be effective in preventing both HIV and HCV.

- Europe is now facing a serious challenge of improving the effectiveness and coverage of its needle and syringe distribution facilities, often due to budget cuts in regard of the economical crisis. In our experience at Fixpunkt, confirmed by experts from other organisations in Germany and within the European wide Correlation network [8], NSP have had limited success for the following reasons: Needles and syringes that the health department provides PWID are often made of inferior quality materials or are not appropriate for their needs. Poor-quality needles, for example, become blunt after one pinprick, and users often need more than one attempt to hit a vein. Program staff members are not able to consult with their clients on the kind of needles or syringes that they need and must accept the standard, poor-quality materials offered by the health department. Health department budget constraints, in addition to the department’s apparent lack of interest in improving the quality of materials used, are among the reasons for this ongoing concern.
- Additional injection equipment to prevent virus transmission (for example, water, filters and spoons) and hygiene products (for example, hand and surface disinfectants and safe disposal boxes for used needles) are often not available.
- Coverage is insufficient because of inadequate budgets and ideological barriers that limit programs’ hours of operation and number of geographical locations (especially in rural or remote areas) and because of other restrictions (for example, only one-to-one exchanges are permitted, and sale of materials is not permitted to under-age individuals).
- NSP are headed by staff members who often do not have adequate knowledge of the issue; funding for training is often limited, and organizations themselves often do not fully appreciate the need for in-depth training. Furthermore, because of insufficient training, many staff members are unable to adequately inform and motivate PWID on how to correctly use prevention equipment and consumption materials in a safe manner and on how to protect themselves from blood-borne infections like viral hepatitis and HIV.
- In most prisons, access to sterile injection equipment is unavailable.
- Police checks and other interventions by law enforcement hinder access to NSP and create fear among PWID who seek NSP services.

A range of effective NSP service modalities can be utilized to complement and reinforce current prevention efforts, including primary NSP and peer-based (secondary) NSP; pharmacy-based syringe vending machines; mobile and outreach services; and distribution in prison settings. Based on our experience at Fixpunkt, effective NSP are:

- Programs that are accepted and actively supported by all key stakeholders (for example, politicians, funders and police);
- Programs that offer high quality materials (needles, syringes, paraphernalia, disinfectants) for preventing transmission of infections;
- Programs that can efficiently provide prophylactic and/or therapeutic motivation to reinforce PWIDs’ ability to take care of their health and avoid (re-) infection being directly (on site) linked to additional interventions (supervised consumption, information and training of drug users)

Neglecting simple preventative measures can have far-reaching consequences. In Berlin, for example, 591 new HCV infections were detected in 2014. If the level of funding of needles, syringes and other sterile or hygienic materials had reached that of the amount spent on only four or five HCV treatments each year, Berlin’s harm reduction services would have been able to reach a much larger number of people.

With such experiences in mind, we, as service providers, propose that, for each euro invested in medical treatment, one cent should be invested in prevention, mainly through the distribution of needles, syringes and other hygienic consumption materials.

#### References

1. European Initiative on Hepatitis C and Drug Use. Manifesto. Berlin Declaration. Hepatitis C: access to prevention, testing, treatment and care for people who use drugs. Berlin, Germany. 23 Oct 2014. http://www.hepatitis-c-initiative.eu/index.php/component/content/article/2-uncategorised/29-manifesto-berlin-declaration. Accessed 10 April 2016.

2. ECDC, EMCDDA. Evidence for the effectiveness of interventions to prevent infections among people who inject drugs. Part1: Needle and syringe programmes and other interventions for preventing hepatitis C, HIV and injecting risk behavior. Stockholm: ECDC.2011.

3. WHO, UNODC, UNAIDS. Technical guide for countries to set targets for universal access to HIV prevention, treatment and care for injecting drug users—2012 revision. Geneva: WHO.2012.

4. WHO. Guidance on prevention of viral hepatitis B and C among people who inject drugs. Geneva: WHO. 2012.

5. Bruggmann P, Grebely J. Prevention, treatment and care of hepatitis c virus infection among people who inject drugs. Int J Drug Policy. 2015;26:S22-S26.

6. European Monitoring Centre for Drugs and Drug Addiction (EMCDDA). European drug report 2015. Luxemburg: Publications Office of the European Union. 2015:69-70. http://www.emcdda.europa.eu/edr2015. Accessed 10 April 2016.

7. UNAIDS. GAP Report 2014. Geneva. 2014:7-9. http://www.unaids.org/sites/default/files/media_asset/05_Peoplewhoinjectdrugs.pdf. Accessed 10 April 2016.

8. Correlation Network. Newsletter 3, policy paper: Who is paying the price for austerity?, Amsterdam, 2013.

